# Diversity dynamics in New Caledonia: towards the end of the museum model?

**DOI:** 10.1186/1471-2148-11-254

**Published:** 2011-09-14

**Authors:** Marianne Espeland, Jérôme Murienne

**Affiliations:** 1 Swedish Museum of Natural History, Entomology Department, Box 50007, 104 05 Stockholm, Sweden; 2 Stockholm University, Department of Zoology, 106 91 Stockholm, Sweden; 3 Department Organismic and Evolutionary Biology and Museum of Comparative Zoology, Harvard University, 26 Oxford Street, Cambridge, MA 02138, USA; 4 Département Systématique et Évolution, UMR 7205, Muséum national d'Histoire naturelle, 45 Rue Buffon, 75005 Paris, France

## Abstract

**Background:**

The high diversity of New Caledonia has traditionally been seen as a result of its Gondwanan origin, old age and long isolation under stable climatic conditions (the museum model). Under this scenario, we would expect species diversification to follow a constant rate model. Alternatively, if New Caledonia was completely submerged after its breakup from Gondwana, as geological evidence indicates, we would expect species diversification to show a characteristic slowdown over time according to a diversity-dependent model where species accumulation decreases as space is filled.

**Results:**

We reanalyze available datasets for New Caledonia and reconstruct the phylogenies using standardized methodologies; we use two ultrametrization alternatives; and we take into account phylogenetic uncertainty as well as incomplete taxon sampling when conducting diversification rate constancy tests. Our results indicate that for 8 of the 9 available phylogenies, there is significant evidence for a diversification slowdown. For the youngest group under investigation, the apparent lack of evidence of a significant slowdown could be because we are still observing the early phase of a logistic growth (i.e. the clade may be too young to exhibit a change in diversification rates).

**Conclusions:**

Our results are consistent with a diversity-dependent model of diversification in New Caledonia. In opposition to the museum model, our results provide additional evidence that original New Caledonian biodiversity was wiped out during the episode of submersion, providing an open and empty space facilitating evolutionary radiations.

## Background

New Caledonia is one of the 10 original biodiversity hotspots [1,2]. Indeed, New Caledonian biodiversity is exceptional for an archipelago of its size (only 19 000 km^2^) [3-5] and also very distinct [6] with a level of endemism of seventy-seven percent at the species level and fifteen percent at the generic level for plants [7,8]. There has been considerable debate about the origin of New Caledonia's tremendous biodiversity [9,10] and molecular phylogenies of extant taxa have provided a useful window into the tempo and mode of species diversification [11,12]. With the growth of phylogenetic studies in New Caledonia [13], we now have a framework to study temporal diversification patterns in the area. Rather than focusing solely on molecular dating techniques, we here investigate how information on diversity dynamics can be used to test the two fundamental models invoked to explain New Caledonian biodiversity.

**Figure 1:**
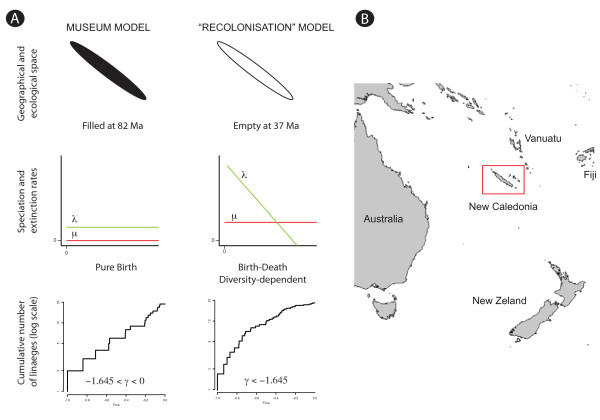
**Expectation under different models**. Location of New Caledonia in the South Pacific (panel B). Different models of diversification expected under different models of the origin of the biodiversity in New Caledonia (panel A). A Pure Birth model where speciation (*λ*) is constant and extinction (*μ*) equals zero is expected under the museum model, corresponding to a Lineage Through Time (LTT) plot closely resembling a straight line. A Birth-Death diversity dependent model is expected under the recolonization model corresponding to a LTT plot showing a slowdown of diversification. Several diversity-dependent models exist and we depict here a model where extinction rate is constant and speciation rate decreases as a function of the number of species.

Due to its Gondwanan continental origin, its long isolation from neighbouring landmasses (like New Zealand or Australia, Figure 1) and its supposed climatic stability, the museum model [14,15] has classically been invoked to explain the origin of New Caledonian biodiversity [16]. According to this classical view, the slow and gradual accumulation of species from ancient Gondwanan stock with low or absent extinction could explain the high level of species richness on the island [10]. Under this scenario, we would expect biodiversity to follow an exponential model of diversification (Figure 1) where per-lineage rates of speciation and extinction have been constant with no upper limit on clade diversity [17,18]. This model specifically refers to the old age of the territory to explain the high level of biodiversity. It is thus based on the premise that species richness is coupled with clade age, meaning that old clades on average have more species than young ones.

As opposed to this classical view, the geology of the territory indicates a complete submersion of the island for 20 Ma (from 65 to 45 Ma) following its breakup from the eastern margin of Gondwana (*c*. 80 Ma) and the presence of an island on the New Caledonia Ridge has only been established since the Late Eocene (around 37 Ma) [19]. All endemic Gondwanan species would have gone extinct and current diversity would have descended from later colonists, whether from nearby island refugia or from long-distance dispersal [10]. This scenario implies a novel ecological space that is open and available, thus facilitating evolutionary radiations [12,20]. Under this 'ecological opportunity' model [21], as time passes and diversification progresses, the geographical and ecological space becomes increasingly saturated with fewer opportunities for speciation [22,23] resulting in a slowdown of diversification rates through time. Under such a scenario, we would expect species diversification to follow a typical niche-filling model [24-26] where the probability of speciation and/or extinction should vary inversely with the number of species, according to a diversity-dependent process [27-30]. We will use the term diversity-dependence [28] rather than density-dependence [31,32] because this process refers to the density of species (diversity) rather than the density of individuals [33].

In this paper, we present the First comparative analysis of species diversification in New Caledonia. Our goal is to estimate diversification dynamic parameters in order to test the two models classically invoked to explain New Caledonia's exceptional biodiversity. We used various groups of angiosperms, lizards, harvestmen, caddisflies and diving beetles, and tested diversification models using the gamma statistic [34] and likelihood models [35].

## Results

Our statistics-based results (Table 1; Figure 1) show that only three groups out of nine did not have a significantly negative *γ*: *Gracilipsodes*, *Helicopha *and *Xanthochorema*, suggesting that for most of the studies, a constant rate could be rejected. All the groups with significantly negative *γ *also passed the MCCR test. A recent study showed that the *γ *test does not necessarily detect early bursts of diversification [36] and that model based approaches might be more appropriate for investigating diversity dynamics. Our model-based results show that only the diversification of one group is best explained by a constant rate model: *Helicopha*. For all the remaining groups, the model-based approach shows a significant positive ΔAIC indicating a better fit to rate-variable models, each time with an inferred slowdown in diversification rates through time. In one case, *Agmina*, a Yule 2-rate process model was inferred as the best-fit model. In all the remaining cases (7 out of 9), the best fitted model was a linear variant of the diversity-dependent model (DDL), though with p = 0.08 for the bayesian analysis of *Papuadytes *and p = 0.06 for the bayesian analysis of *Gracilipsodes*. More complex models (SPVAR, EXVAR and BOTHVAR) allowing a non-zero probability of extinction did not provide a better fit. For our studies, there is also a clear decoupling between clade age and clade size (Pearson's r = 0.22).

**Table 1:** Age and parameter estimates of the diversification analyses.

Parameter estimates														
						Pure birth model			DDl/Yule 2-rate					
Dataset	Age estimate	Method	γ	γ_C_	LH	AIC	r1	LH	AIC	r1	r2	k	st	ΔAIC (DDL-pb)
*Niemeyera*	32.4 Ma	Penalized likelihood	-2.219 (p = 0.013)	-1.631 (p = 0.013)	104.013	-206.026	2.312	107.184	-210.368	4.479	N/A	52.716	N/A	4.342 (p = 0.039)
		Bayesian inference	-2.348 (p = 0.009)	-1.638 (p = 0.009)	132.285	-262.569	2.678	135.736	-267.472	5.080	N/A	61.432	N/A	5.278 (p = 0.035)
Scincidae	35.4-40.7 Ma	Penalized likelihood	-4.311 (p = 8.12e-06)	-1.793 (p = 2.00e-04)	83.371	-164.742	1.745	94.684	-185.368	5.808	N/A	39.262	N/A	20.626 (p = 0)
		Bayesian inference	-3.752 (p = 8.79e-05)	-1.811 (p = 2.00e-04)	93.171	-184.342	1.907	101.696	-199.392	5.375	N/A	42.899	N/A	15.050 (p = 0.001)
Troglosironidae	49 Ma	Penalized likelihood	-2.951 (p = 0.002)	-1.299 (p = 4.00e-04)	12.658	-23.317	1.396	19.896	-35.792	8.555	N/A	12.339	N/A	12.476 (p = 0)
		Bayesian inference	-2.173 (p = 0.015)	-1.324 (p = 0.006)	17.666	-33.332	2.201	21.667	-39.334	8.796	N/A	12.778	N/A	6.003 (p = 0.011)
*Papuadytes*	9 Ma	Penalized likelihood	-1.943 (p = 0.026)	-1.518289 (p = 0.019)	18.348	-34.697	1.606	21.468	-38.936	5.018	N/A	15.474	N/A	4.239 (p = 0.04)
		Bayesian inference	-1.648 (p = 0.050)	-1.554558 (p = 0.041)	19.569	-37.137	1.764	21.294	-39.882	4.865	N/A	16.024	N/A	2.745 (p = 0.086)
*Gracilipsodes*	14.4 Ma*	Penalized likelihood	-1.082 (p = 0.140)	-1.325 (p = 0.081)	7.671	-13.342	1.431	9.201	-14.403	4.307	N/A	10.309	N/A	1.061 (p = 0.045)
		Bayesian inference	-1.596 (p = 0.108)	-1.309 (p = 0.059)	7.535	-13.070	1.407	9.357	-14.714	4.600	N/A	10.088	N/A	1.644 (p = 0.062)
*Helicopha*	8.2 Ma*	Penalized likelihood	-0.491 (p = 0.312)	-1.345 (p = 0.229)	32.733	-63.466	2.191	33.152	-62.303	3.301	N/A	32.985	N/A	-1.162 (p = 0.563)
		Bayesian inference	-0.016 (p = 0.404)	-1.399 (p = 0.400)	24.042	-66.083	2.367	34.137	-64.274	2.906	N/A	56.686	N/A	-1.809 (p = 0.828)
*Xanthochorema*	11.9 Ma*	Penalized likelihood	-1.598 (p = 0.055)	-1.309 (p = 0.028)	7.726	-13.453	1.441	10.446	-16.891	5.858	N/A	9.688	N/A	3.437 (p = 0.026)
		Bayesian inference	-1.613 (p = 0.053)	-1.305 (p = 0.023)	7.718	-13.436	1.440	10.479	-16.958	5.906	N/A	9.676	N/A	3.522 (p = 0.016)
Hydropsychinae	28.2 Ma	Penalized likelihood	-2.850 (p = 0.002)	-1.516 (p = 0.002)	57.944	-113.888	2.108	64.609	-123.219	3.185	0.417	N/A	0.176	9.33118 (p = 0)
		Bayesian inference	-2.582 (p = 0.005)	-1.519 (p = 0.004)	58.046	-114.091	2.116	62.474	-120.949	5.387	N/A	30.960	N/A	7.933 (p = 0.007)
*Agmina*	21.9 Ma	Penalized likelihood	-2.647 (p = 004)	-1.804 (p = 0.007)	242.743	-483.486	2.544	250.947	-495.894	7.539	2.118	N/A	0.639	12.40858 (p = 0.001)
		Bayesian inference	-1.969 (p = 0.024)	-1.885 (p = 0.043)	249.704	-497.407	2.799	255.126	-504.252	6.755	2.404	N/A	0.596	6.845 (p = 0.153)

Age estimates followed by an asterisk indicate those calculated in this publication, other estimates are taken from the literature (see text). In the DDL/Yule 2-rate column parameters are for the DDL model for all groups except the *Agmina *where Yule 2-rate parameters are shown. *γ *is the diversification statistic by [34], *γ*c is the threshold required for the *γ *still to be significant after accounting for missing taxa (MCCR test), LH is the maximum likelihood of the model, AIC is the Akaike information criterion, r1 and r2 are diversification rates, k is the carrying capacity, st is the rate shift point, ΔAIC is the di**ff**erence in AIC between the pure birth (Yule model) and the rate variant model.

## Discussion

### Biases towards an apparent slowdown

Diversification analyses are sensitive to biases in the phylogenetic reconstruction method [37]. For example, multiple substitutions could lead to saturation of genetic distances producing incorrectly short branch lengths deeper in the tree [38]. We chose the GTR model because it is the most common and general model for real world DNA. While many authors have used the GTR + I + Γ to incorporate rate heterogeneity [39], it is well established [40] that adding a proportion of invariable sites creates a strong correlation between p0 (parameter of I) and *α *(parameter of Γ), making it impossible to estimate both parameters reliably [41,42]. Following RAxML's manual recommendations, we used a GTR + Γ model [40] applied to each partition. This should ensure that our results are not biased by under-parameterization of our phylogenetic reconstruction.

The method of phylogenetic ultrametrization could also influence the estimate of *γ *[43-45]. In this study we used two different methods: First, maximum likelihood (with RAxML for phylogenetic inference and R8S for ultrametrization) and secondly, bayesian inference (with BEAST). R8S uses an autocorrelated relaxed molecular clock while BEAST uses a non-correlated clock. This should ensure that our results (which were consistent whichever method was used) are not biased by ultrametrization techniques. Incomplete taxon sampling can also introduce some bias in favor of a pattern of slowdown of diversification rates and towards a more negative *γ *[34,46]. If the sampling is incomplete, the critical value must be adjusted. Our results were not biased by taxon sampling as indicated by the result of the MCCR test. It is worth noticing that we were very conservative when conducting the MCCR test by adding an extra 10% to the known unsampled diversity.

If the diversification of a group follows a Yule process and the sampling is apparently complete, a bias towards slowdown can still exist if recent lineage splits are unlikely to be considered as distinct species. Indeed, recent lineage splits are likely to be recognized as speciation event only if both lineages persist long enough to evolve differences that attract taxonomic attention [47]. Population-level studies are still scarce in New Caledonia [48-51] but a growing trend has been to include several specimens of the putative same species in phylogenetic reconstruction [11]. In particular, several phylogenies included in the present study have used multiple specimens from the same species that we here considered as separate entities [12,52,53] The previous situation is close to the case of non-random sampling where systematists tend to oversample deep nodes to get a better coverage of the taxon's morphological diversity [54], later referred to as 'diversified sampling' [55]. Studies in New Caledonia are usually performed with the aim of inferring biogeographic evolutionary history rather than simply reconstructing the systematics of the group. In addition, most of our datasets have a relatively low number of missing taxa and in all cases, the proportion of sampled species is more than the 80% level recently proposed as a threshold [54,56].

A pattern of slowdown in large clades is expected under constant speciation-extinction models, whenever the extinction rate is low [57]. Due to stochasticity, large clades (produced if, by chance, multiple speciation events happened early in the diversification) and small clades (produced if, by chance, few speciation events happened early in the diversification) will both tend to go back to the average speciation rate following a regression effect. Under this situation, we expect to see an apparent slow down in large clades and acceleration in small clades. Our results are not consistent with this situation as the largest diversification (*Agmina*) shows a relatively low *γ *while Troglosironidae, with only 11 species, shows one of the highest negative *γ*.

As explained recently [58], negative *γ *can also be achieved if a clade is in significant decline. There are numerous examples from the fossil record showing clades in decline [59] and recent taxa that have become extinct can only be assessed using the fossil record. Unfortunately, in many cases (especially in New Caledonia), there is no good fossil record and molecular phylogenies cannot infer declines. The average rate of diversification needed to account for the living diversity may have nothing to do with the actual diversity trajectory that led to the living diversity. As for all the studies on diversity dynamics, the absence of information from the extinct species is a severe limitation [58].

### Towards a rejection of the museum model?

The museum model makes the assumption that there is a strong correlation between clade age and species richness. Our results show evidence for diversification slowdown suggesting that diversification might be diversity-dependent. In addition, seven of the nine datasets present are better explained by a diversity-dependent model than alternative models of diversification. This pattern of evolution has long been observed in the fossil record [60-62]. This general model also explains why many studies have shown that clade age and clade size are not related [63-65]. For our studies, there is also an evident decoupling between clade age and clade size (Pearson correlation factor r = 0.22).

Constant birth and extinction rates produce an apparent increase in diversification rates on the reconstructed phylogenies. Under this scenario, we would expect a positive *γ *[34]. This indicates that our results broadly reject a constant rate diversification process, whether diversifications followed a Pure Birth or a Birth-Death model. However, if clades are too young, we could observe an apparent absence of diversity regulation that results from insufficient time to reach carrying capacity [66]. Recent simulation studies have indeed demonstrated that during the early phase of a logistic growth, the *γ *statistic is unable to identify a diversity-dependent process [67].

Our results based on the diversity dynamics of the phylogenies reject the museum model. They also agree with molecular dating results, rejecting the hypothesis that the distribution of New Caledonian groups could be explained by vicariance from Gondwana around 80 Ma [13]. Perhaps the most unusual case is the New Caledonian endemic harvestman family Troglosironidae, sister to the Tropical Gondwanan family Neogovidae [68]. The start of the diversification of the group was estimated at 28-49 MYA in [68]. The age of the same group is currently estimated to be around 77 Ma ('much pre-dating the total submersion episode that would have ended by 37 Ma') but this study only included two species [69]. This group has consistently been presented as a Gondwanan relict whose presence in New Caledonia had to be explained by vicariance [70] even though confidence intervals [68,69] are also consistent with a more recent dispersal scenario. Our results indicate that the diversification of Troglosironidae in New Caledonia show the same characteristics as other diversifications (e.g. *Niemeyera*), suggesting a diversity-dependent process of diversification after recolonization.

### Alternative Gondwanan models?

In this paper, we specifically tested a model of biodiversity (the museum model) where species with a Gondwanan origin in New Caledonia would have evolved following an exponential model (implying constant rates of speciation and extinction (if any) in a stable environment). Alternatively, we could imagine additional models where the New Caledonian clades still take their origin from a vicariant event due to the fragmentation of Gondwana.

**Figure 2:**
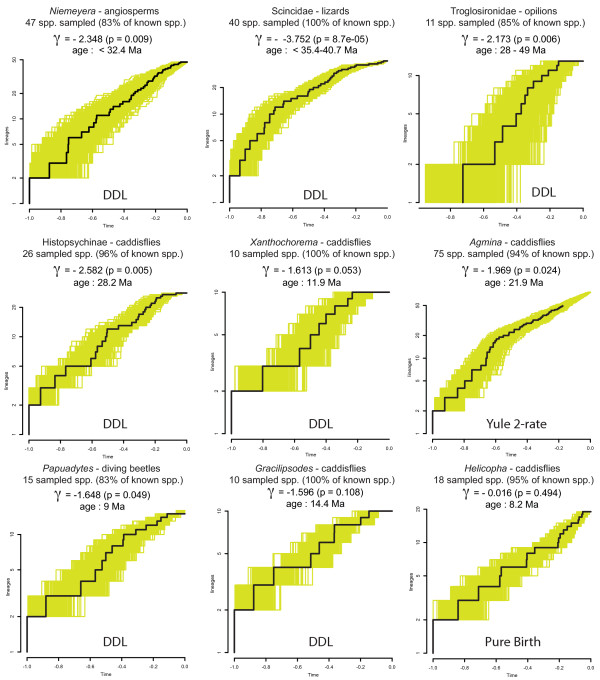
**Results**. Lineage through time (LTT) plots observed for 1000 trees drawn randomly from the posterior distribution of the Beast analyses (plotted in yellow) to get a measure of uncertainty in our estimates. *γ *was calculated based on the consensus tree. For each group, the number of taxa included is indicated (see text for details) as well as the proportion of known diversity included. The best-fit model is indicated below each graph (DDL: diversity-dependent linear).

Under a hypothesis of a constant speciation/extinction rate process (as in the museum model), we could also imagine that an event of mass extinction occurred. This model results in LTT plots showing antisigmoid curves with a distinct signature (a 'broom and handle' shape) [71]. They rise steeply at first, curve to a plateau and rise steeply again to the present. Even though those LTT plots were shown to be indistinguishable from those produced by a model where rates are constant but interrupted by a phase of stasis [72], they are still very different from those produced by typical diversity-dependent models such as in our results (Figure 2).

Another alternative is where the New Caledonian original stock from Gondwana would have evolved following a diversity-dependent model. What are the expectations of such a model? It was originally suggested that a signature of diversity-dependence could only be observed if extinction was zero [73]. However, further simulation studies demonstrated that substantial extinction can occur without erasing the signal of an underlying decreasing diversification rate provided the initial speciation rate is high enough [74]. It is in fact the ratio between the initial speciation rate and the rate of extinction at equilibrium (the so-called 'LiMe ratio') that is critical in determining the shape of the phylogeny. For low values of LiMe, diversity-dependent diversification produces phylogenies that are indistinguishable from those expected under sustained and constant rates of diversification [67].

There is strong evidence for an initially high speciation rate in association with ecological opportunity, both in the case of mass extinction [75] and colonization of recent islands [20,76]. In the case of an already filled geographical and ecological space, as it is hypothesised in our alternative Gondwanan model, we would expect speciation rates to be relatively low. In this case, the low LiMe value would erase the diversity-dependent signal and we would expect *γ *values to be non-significant.

Even if we imagine that the LiMe ratio was sufficiently high to produce a pattern of diversity-dependence, another issue remains. Considering that New Caledonia broke off from the Eastern margin of Gondwana a long time ago (around 80 Ma), under a model of diversity-dependence, we would expect most of the clades to have reached their equilibrium and entered a state of species turnover at constant diversity. Recent simulation studies have demonstrated that in the case of a diversity-dependence process with high LiMe ratio, there is only a short temporal window where the *γ *statistics can detect a diversification slowdown. Shortly after the equilibrium is attained, the average *γ *becomes indistinguishable from the null model of a constant diversification rate [67].

Under our alternative Gondwanan scenario, we would expect to observe constant rates of diversification and no apparent slowdown. This is either related to the low LiMe ratio or the fact that clades have long reached their equilibrium. Considering that our results show evidence of slowdown in New Caledonian diversifications, we also reject this alternative model.

### Impact of biotic interactions

It was recently argued that 'ancient radiations' or 'repeated dispersals' were two opposing explanations for New Caledonian biodiversity [13,77]. This simplistic view is however confusing since 'repeated dispersal' represents a pattern while 'ancient radiation' implies a process of diversification. The two elements are thus not necessarily in opposition. Our results indicate that in several cases, evidence of an early burst of diversification (consistent with a radiation process) is observed, even though multiple dispersal events have been inferred.

In the case of *Niemeyera *[78], based on pairwise genetic divergence and the slowest rate available, the authors previously concluded [78] that the oldest divergence between sister Australian and New Caledonian taxa might have taken place approximately 32.4 MA. They also showed that two other groups of Sapotaceae are present on the island. Here, we studied the oldest diversification which diversified substantially. In the case of caddisflies, it seems that the diversification of a young clade (*Caledomina*) with few species has been impeded by competition with the closely related older *Agmina *extremely diverse radiation [79].

An alternative example is given by the case of the diving beetles, *Papuadytes*. The group is absent from Fiji where the genus *Copelatus *diversified extensively [80], partly occupying the same habitats as *Papuadytes *in New Caledonia. Conversely, *Copelatus *is absent in New Caledonia suggesting that competition between groups is an important factor in explaining their distribution. In contrast to the *Niemeyera *example, the older New Caledonian *Papuadytes *group (c. 14 Ma) has only two species representing relictual species pushed to marginal habitats (high altitudes) by subsequent arrivals (c. 9 Ma) [81].

These examples clearly highlight the role of interspecific competition, providing further evidence for the finding that diversification processes in New Caledonia follow a diversity-dependent model.

### Role of ecology and geography

Early bursts followed by a slowdown in diversification are usually interpreted within a framework of adaptive radiation [79]. However, the notion of adaptive radiation [60,82] specifically refers to the evolution of ecological and phenotypic diversity within a rapidly multiplying lineage [20], and thus not just to a pattern of temporal variation in diversification rates. Coarse-grained niche modeling studies have shown that climate variables are broadly similar among related species [83,84]. However, few examples show that differences in fine-scale micro-habitat exist in groups that are otherwise similar in their climatic requirements [11,80]. There are very few studies investigating the rate of trait evolution through time [33,85-88] and none of the New Caledonian studies have addressed this critical issue. It is thus apparent that the adaptive nature of the New Caledonian radiation is far from being established. In a neutral setting, allopatric speciation related to vicariance will result in a split of the ancestral geographical space [89-91]. The newly created restricted ranges will likely influence further diversification rates because speciation and extinction rates are related to the species range [92]. A recent simulation study has shown that slowdown in diversification rates can be related to a purely geographical process [93].

The reduction of range size related to speciation will, in turn, lower the probability of speciation, leading to a temporal slowdown in diversification rate. Under this scenario, it is the reduced geographical opportunity (rather than the ecological opportunity) that is responsible for the slowdown. It is also worth noting that the elongated shape of the main island of New Caledonia (with a high ratio of the long to the short axis) is likely to influence speciation probabilities because linear distributions are more likely to be bisected by geographic barriers. In this context, radiations would be non-adaptative [94-96] and deciphering the adaptive nature of New Caledonian radiation becomes, once again, a critical issue.

## Conclusions

In this paper, we reanalyzed all the molecular datasets for New Caledonia that were suitable for our purposes. We reconstructed the phylogenies using standardized methodology, used two ultrametrization alternatives, and took into account phylogenetic uncertainty as well as incomplete taxon sampling when conducting diversification rate constancy tests. Our results provide evidence that the New Caledonian diversifications follow a process of diversity-dependence. This model is consistent with the geological history that indicates a complete submersion of the territory after its breakup from Gondwana [19]. The island was established around 37 Ma, providing an empty geographical and ecological space facilitating evolutionary radiations.

Despite a growing number of phylogenetic studies investigating patterns and timings of diversifications in New Caledonia, the adaptive or non-adaptive nature of those radiations, including their related phenotypic divergences, remains largely unknown. In addition, the influence of phylogenetic niche conservatism/evolution, both at a large (climatic) and fine (habitat) scale needs to be further evaluated [83,84]. Our approach could be applied to other continental islands such as New Zealand and Madagascar where similar debates about the origin of their biodiversity have emerged [97,98].

## Methods

### Datasets

Our selection of datasets was based on clear criteria. Phylogeography studies were not included as branching patterns do not correspond to speciation events [50]. Phylogenetic analyses had to be based (at least partly) on molecular data. We thus discarded all the published phylogenies only based on morphological data [84]. Species diversification in New Caledonia had to be substantial to be incorporated (at least ten species). We thus discarded all the published phylogenies presenting small diversifications [77,99], including only few species from New Caledonia [100,101] or only performed at the genus level [102]. The original dataset had to show phylogenetic resolution among the New Caledonian species. We thus discarded all the published phylogenies with little to no resolution [103-105]. Finally, the dataset had to include all the species (or at least most of the species) of a monophyletic New Caledonian group. We discarded studies [11,106] that included only part (one genus) of monophyletic groups in New Caledonia [107] or paraphyletic New Caledonian groups [108,109]. All the included members could be interpreted as classical Gondwanan groups based solely on their distribution.

The ***Niemeyera *dataset **[52] consists of a monophyletic group (the "*Niemeyera *complex" of the Sapotaceae subfamily Chrysophylloideae) of 47 species (36 known species with several undescribed species) sister to a group of Australian species. Eight species for which material was unavailable were not included in the study. Three accessions of *Pycnandra fastuosa *were included in the study. The results were not conclusive regarding the monophyly of the species and the branch lengths were also longer than between member of different species. For these reasons, we kept in our analyses those three accession as separate entities.

The **Scincidae dataset **[53] includes 42 species representing all the recognized species of Scincid lizards of New Caledonia except five. The Tasmantis (Zealandia) clade was found monophyletic but not the New Caledonian species. We used in the present study the larger monophyletic diversification in New Caledonia. The two specimens of *Nannoscincus gracilis, Caledoniscincus austrocaledonicus *and *Nannoscincus mariei *were kept as separate entities as they do not form a monophyletic group.

The **Troglosironidae dataset **[70] consists of a monophyletic group of 11 species of harvestmen (among the 13 species known from New Caledonia) representing the full geographical range of the group. All of the species are endemic and comprised in one endemic genus in one endemic family. The Troglosironidae study [70] was based on direct optimization [110] and it was thus necessary to reanalyze the dataset.

For the ***Papuadytes *dataset **[81], the authors concluded that lineages of those diving beetles colonized New Caledonia twice, around 14 and 9 Ma (for the larger diversification), and both lineages are derived from an Australian ancestor. We included 15 species of the larger diversification which is currently estimated at 18 species.

The ***Gracilipsodes *dataset **[111] is a New Caledonian endemic genus of caddisflies (Trichoptera) in the family Leptoceridae. The genus at present consists of 10 species and is sister group to the eastern Australian monotypic genus *Triplexa*. No dating is available for this dataset.

The ***Helicopha *dataset **[112] is a monophyletic genus of caddisflies in the family Helicophidae with currently 18 described species, of which 17 are included in the dataset in addition to two still undescribed species. Four members of the genus are also found in Australia. No dating is available for this dataset.

The ***Xanthochorema *dataset **[113] is a monophyletic New Caledonian endemic genus (9 described and one undescribed species) of caddisflies with free-living predatory larvae in the family Hydrobiosidae [114]. The sister group *Psilochorema *is found in New Zealand.

The **Hydropsychinae dataset **[12] showed that there was only a single diversification of Hydropsychinae caddisflies in New Caledonia and not three as previously thought. This radiation consists of 27 described species of which 26 are included in the dataset. Additionally three specimens of the species *Caledopsyche atalanta *and two specimens of *Orthopsyche nadauna *are included since the branch lengths between these specimens are longer than between different species. The total dataset therefore includes 28 species in total. An age of approximately 28.2 Ma has been estimated for this group [12].

The ***Agmina *dataset **consists of a monophyletic endemic diversification of caddisflies with at least 80 species (only 27 are presently described), of which 75 are included in the phylogeny [79]. This is the largest animal diversification known from New Caledonia. *Agmina *split off from its Australian ancestor around 36,6 Ma (CI: 29.7-48.3 Ma) ago and the New Caledonian radiation started approximately 21.86 Ma (CI 16.8-24.6 Ma). In the same family (Ecnomidae) there is a second New Caledonian endemic genus (*Caledomina*) with only 4 known species, which split from its Australian ancestor 25,9 Ma (CI: 21.4-38.2 Ma) and started diversification around 9.5 Ma (CI: 6.4-13.2 Ma). The latter is not included in our analyses.

### Diversity dynamics

#### Phylogenetic inference and dating

Most of the studies previously presented used a variety of alignment and analyses strategies rendering the results difficult to compare or impossible to further analyze. When alignments were not provided by the authors or when Direct Optimization [115] was previously used, sequences were downloaded from GenBank. All the source phylogenies were reanalyzed for this study. Alignment was performed with MUSCLE 3.6 [116] most accurate algorithm and variable regions were removed using GBLOCK 0.91b [117] with options -t = d -b5 = h. Concatenation of the genes fragments was performed with PHYUTILITY[118]. When clade ages were not available from the original dataset, we estimated the diversification age (the age of the most common recent ancestor of the group) based on the COI gene with a 2.3% pairwise divergence per million years [119] calculated with PAUP[120]. It has been shown that this standard Arthropod molecular clock is not always correct [121], but since age determination was not the main goal of this paper, we included this approach in order to get a rough estimate of the timing of diversification. Pearson's r was calculated to estimate the degree of correlation between clade ages and sizes.

For the maximum likelihood analyses, phylograms were computed using RAxML 7.0.4 [122] with a GTR + Γ model [123] applied to each partition. Chronograms (i.e. phylogenies with branch length proportional to time) were estimated using standard likelihood methods as implemented in the program R8S 1.71 [124,125]. We used a cross-validation procedure [126] to select the best method among those offered by the program. We tested one clock-like method, the Langley and Fitch method [127], and two relaxed-clock methods, nonparametric rate smoothing [128] and penalized likelihood [126]. For the penalized likelihood method, the degree of autocorrelation within lineages was estimated using cross-validation, and the smoothing parameter *λ *defined accordingly. We also tested the performance of two penalty functions, the additive penalty function, which penalizes squared differences in rates across neighboring branches in the tree, and the log penalty function, which penalizes the squared difference in the log of the rates on neighboring branches. A search was then performed using the commands num_time_guesses = 3 (3 initial starting conditions) and check-Gradient in order to validate the results. After pruning the outgroups, all trees were calibrated to a total depth from root to tip of 1.

Bayesian analyses were performed using BEAST 1.5.2 [129] as run on the BIOHPC cluster at Cornell University. We performed two separate runs of 20 million generations with sampling every 1000 generations. For all datasets, the analyses were run under a GTR + Γ model for each partition, using a lognormal relaxed clock. All priors were kept as default except the tree prior which was set to a Yule model and the age of the root of the New Caledonian radiation of interest which we constrained to one using a normal distributed prior with mean 1 and standard deviation of 1× 10-7 (thus roughly corresponding to the calibration used in R8S). Convergence was assessed in TRACER 1.5 [130] and the two runs were thereafter combined in LOGCOMBINER 1.5 in the BEAST package, after removing the First 8000 samples of each run as burn-in. For the *Agmina *dataset two runs of 40 million generations were performed to ensure convergence.

#### Diversification analyses

The linearity for the relationship of log(number of lineages) against time can be visually inspected with a Lineage Through Time plot [131,132]. If diversification has been constant through time, then a straight line is expected. In addition, the *γ *test [34] reduces the information available in a molecular phylogeny to a single number, which provides insight into whether the net rate of diversification decreased over time. The *γ *statistic describes the center of mass for the nodes in a chronogram. Under a pure birth model, this statistic follows a normal distribution with mean = 0 and standard deviation = 1. For a given phylogeny with no missing taxa, a constant rate of diversification is rejected if *γ *<*−*1.645 (type I error probability *α *= 0.05, one tailed) and nodes are more concentrated towards the base of the clade.

The Lineage Through Time (LTT) plots [131,132] and *γ *statistic [34] were computed with APE[133]. For the chronogram obtained under bayesian analysis, we calculated the *γ *statistic for the maximum clade probability tree with mean node heights. The *γ *statistic was calculated for 1000 trees drawn randomly from the posterior distribution to get a measure of uncertainty in our estimates. The *γ *statistic assumes complete taxon sampling, which is not true (or probably not true) for our phylogenies. Because incomplete taxon sampling could bias the estimates of *γ *[34,134], we conducted a Monte Carlo constant rates test as implemented in LASER with 5000 replicates. Phylogenies were simulated to the hypothetical full clade size (known missing taxa + 10% unknown missing taxa) under a constant rate pure birth diversification process and taxa were randomly pruned from the tree to mimic incomplete sampling.

Finally, probabilistic models [135] were evaluated by maximum likelihood in order to investigate more complex temporal patterns of diversification. Diversification parameters were computed using the best-fitting model among two rate-constant (Yule 1-rate and birth-death model) [131,136] and three rate-variable diversification models (Yule 2-rate, diversity dependent linear, diversity dependent exponential) [35]. P values were calculated by generating 1000 trees under a Yule model assuming 10% missing taxa in addition to the known non-sampled taxa as indicated in the original article. When decreases in diversification rates are observed, new models can be used for explicitly parsing out the relative importance of changes in speciation and extinction rates [73,74,137,138]. The first model of diversification (SPVAR) is characterized by an exponential diversity-dependent speciation rate and a constant extinction rate [139,140]. The second model (EXVAR) uses a constant speciation rate and linear diversity-dependent extinction. The third model (BOTHVAR) uses linear diversity-dependence for both speciation and extinction rates [24]. Model fitting was conducted with LASER[141]. Model selection was performed by calculating the difference in the Akaike Information Criterion [142] score (ΔAIC) between the best rate-constant and the best rate-variable models [35].

## Authors' contributions

Both authors equally contributed to the study. JM performed the maximum likelihood analyses. ME performed the bayesian analyses. Both authors read and approved the final manuscript.
